# Modeling of suppression and mitigation interventions in the COVID-19 epidemics

**DOI:** 10.1186/s12889-021-10663-6

**Published:** 2021-04-14

**Authors:** Yuexing Han, Zeyang Xie, Yike Guo, Bing Wang

**Affiliations:** 1 School of Computer Engineering and Science, Shanghai University, Shanghai, People’s Republic of China; 2 Shanghai Institute for Advanced Communication and Data Science, Shanghai University, Shanghai, People’s Republic of China; 3 Hong Kong Baptist University, Hong Kong, People’s Republic of China; 4 Department of Computing, Imperial College London, London, United Kingdom

**Keywords:** COVID-19, Basic reproduction number, Interventions, Suppression, Mitigation

## Abstract

**Background:**

The global spread of the COVID-19 pandemic has become the most fundamental threat to human health. In the absence of vaccines and effective therapeutical solutions, non-pharmaceutic intervention has become a major way for controlling the epidemic. Gentle mitigation interventions are able to slow down the epidemic but not to halt it well. While strict suppression interventions are efficient for controlling the epidemic, long-term measures are likely to have negative impacts on economics and people’s daily live. Hence, dynamically balancing suppression and mitigation interventions plays a fundamental role in manipulating the epidemic curve.

**Methods:**

We collected data of the number of infections for several countries during the COVID-19 pandemics and found a clear phenomenon of periodic waves of infection. Based on the observation, by connecting the infection level with the medical resources and a tolerance parameter, we propose a mathematical model to understand impacts of combining intervention measures on the epidemic dynamics.

**Results:**

Depending on the parameters of the medical resources, tolerance level, and the starting time of interventions, the combined intervention measure dynamically changes with the infection level, resulting in a periodic wave of infections controlled below an accepted level. The study reveals that, (a) with an immediate, strict suppression, the numbers of infections and deaths are well controlled with a significant reduction in a very short time period; (b) an appropriate, dynamical combination of suppression and mitigation may find a feasible way in reducing the impacts of epidemic on people’s live and economics.

**Conclusions:**

While the assumption of interventions deployed with a cycle of period in the model is limited and unrealistic, the phenomenon of periodic waves of infections in reality is captured by our model. These results provide helpful insights for policy-makers to dynamically deploy an appropriate intervention strategy to effectively battle against the COVID-19.

## Background

The COVID-19 pandemic has become a major global threat for human lives. In the absence of vaccines, effective treatment, and with limited knowledge of the virus [1–4], non-pharmaceutic interventions have been adopted to slow down the disease propagation. Nations around the world implemented a number of containment policies aimed at mitigating the epidemic. With the progression of the epidemic, individuals have improved their awareness of infection and changed their behavior to reduce their risk of infection by wearing face-masks and washing hands frequently [5]. Policies such as lockdown of the city [6, 7], travel restrictions [8–10], school closure [11], quarantine [12, 13] or stay-at-home [14], social distancing [15–19], bans on gatherings of more than a number of people, tracking individuals who are potentially infected [20, 21] have been implemented to reduce the contact rate and halt the epidemic. Some countries like Singapore used contact tracing to efficiently slow down the epidemic, while other countries such as the UK, opted to herd immunity and then changed to a strict lockdown.

Policy-makers are confronted with difficult choices for controlling the epidemic. On one hand, strict measures on suppressing the epidemic can save people’s lives from deaths, while likely increasing the risk of economical losses; on the other hand, gentle mitigation interventions can reduce negative economical impacts but risk people’s life. Hence, it is necessary to estimate the epidemic dynamics in order to implement efficient, economical interventions accordingly [22, 23]. Many studies have developed mathematical models to evaluate the role of restriction measures on the dynamics of the COVID-19 pandemic [13, 23–28]. Most of previous studies focused on modeling and estimating the transmission rate *β* to reflect the impact of interventions [20, 29]. For instance, a two-step control strategy relating suppression and mitigation was proposed [13, 23] based on modeling. Optimal control of the COVID-19 from the point view of economics was also studied in [25]. The impacts of several interventions including case isolation, voluntary home quarantine, social distancing, and closure of schools have been explored in Ref. [26]. It shows that the most effective combination of interventions in reducing deaths and intensive care unit (ICU) capacity is a combination of case isolation, home quarantine, and social distancing. In a recent study [30], an adaptive intervention strategy was proposed for the control of the COVID-19 epidemic by considering individuals’ mobility between cities. The authors assumed that intervention measures change the transmission rate with a controlled parameter and the floating population. In [24], strategies of intervention combination of suppression and mitigation were compared among 16 countries, while the cycle of the intervention is set at a fixed period. Although intensive interventions can effectively reduce the transmission rate, the epidemic may rebound if interventions are relaxed [26].

Since the epidemic is highly dynamic, rapidly changing with the increase of infection cases, appropriate interventions should be responsive to the outbreak and change with the epidemic dynamics accordingly. To capture this aspect, in this work, we explore a standard epidemiological model modified by considering two types of interventions, i.e., suppression and mitigation, which are dynamically implemented by considering factors, such as the city’s medical resources and its tolerance for infection. We consider that mitigation interventions are a combination of measures, such as general social distancing measures, hygiene rules, case isolation, shielding of vulnerable groups, school closures, etc, targeting the basic reproduction number approaching but larger than 1, while suppression interventions are taken as additional measures of strict physical distancing, including lockdowns, targeting the basic reproduction number less than 1. We propose a combined strategy of suppression and mitigation to control the disease propagation. Instead of setting the cycle of the strategies as a fixed period, the system is allowed to dynamically adjust the control strategy depending on the infection level. By doing so, the epidemic is under control below an acceptable level and a strict suppression intervention is not necessarily deployed during the period.

## Methods

**Fig. 1:**
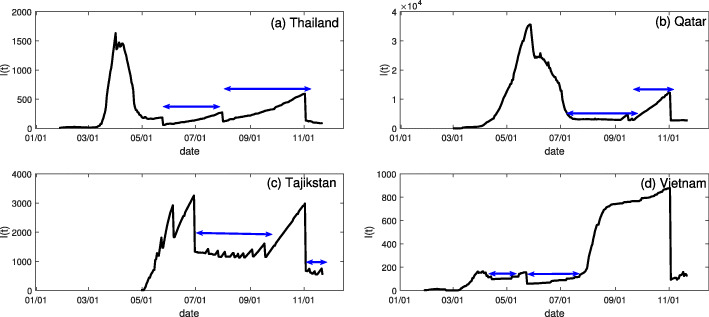
The number of existing confirmed cases in some typical countries. **a** Thailand; **b** Qatar; **c** Tajkstan; **d** Vietnam. The arrow in each panel indicates the plateau period due to the interventions being introduced

In order to understand the impact of different interventions on controlling the epidemic, we firstly collect the data from data source DXY [31] and observe the curves of existing infections in different countries, as shown in Fig. 1. We clearly see that in some countries, the number of existing infections shows a typical, nonuniform periodic wave with different peaks and periods. For instance, in Thailand (Fig. 1a), the number of existing infections reaches the peak around 1500 cases on April, 1st, 2020, and decreases to 100 cases soon before May, 25th, 2020. Then, it follows a period wave of infections less than 200 cases. Recently, it increases to 500 cases again. Also in Qatar (Fig. 1b), during the period from July 12th, 2020 to Sep. 11th, 2020, the number of infections was controlled less than 4000 cases and then it decreases again from Nov. 1st, 2020. Similar phenomena can also be found in Tajikstan and Vietnam (Fig. 1c and d). Although the periods of the waves and the peak values of infections may be different, such a phenomenon of period waves indeed reflects various interventions deployed by nations to battle against the COVID-19 pandemic.

**Fig. 2:**
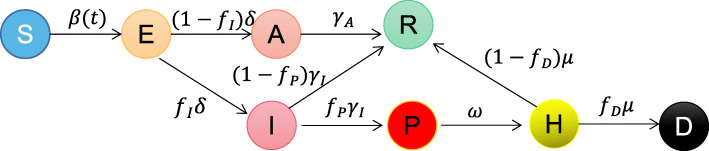
The flow diagram of the compartmental model. Host states are indicated by circles and transitions are indicated by arrows

In the following, we perform a modeling study to understand interventions for the appearance of periodic waves of infections. Since the COVID-19 can cause infections with no symptoms [32] and severely infected individuals have to be hospitalized for treatment, we model them in a population based on the classical susceptible-exposed-infected-recovered (SEIR) epidemiological model, where individuals belong to eight states: susceptible (*S*), exposed (*E*), asymptomatic (*A*), symptomatic (*I*), severe infection (*P*), hospitalized (*H*), dead (*D*), and recovered (*R*). See Fig. 2. The total population size is denoted as *N*=*S*+*E*+*A*+*I*+*P*+*H*+*D*+*R*. At time *t*, susceptible individuals get infected by having contacts (asymptomatic, symptomatic or severe) infectious individuals and move to exposed state (*E*) at a rate *β*(*t*), where *β*(*t*) varies with time due to the involvment of interventions. The infectiousness of asymptomatic and severe infected individuals compared with symptomatic infected individuals are adjusted by factors *θ*_*a*_ and *θ*_*p*_, respectively. A fraction of exposed individuals (*f*_*I*_) moves to symptomatic infectious state (*I*) at rate *δ*, while the remainder of them (1−*f*_*I*_) moves to asymptomatic infectious class (*A*) at the same rate *δ*. Thus, the $\frac {1}{\delta }$ refers to as incubation period. The infectious periods of symptomatic and asymptomatic infections are denoted as $\frac {1}{\gamma _{I}}$ and $\frac {1}{\gamma _{A}}$, respectively. A fraction of symptomatic infections (*f*_*P*_) develops to be severely infected by moving to severe infection (*P*) at rate *γ*_*I*_, while the remainder of symptomatic infections (1−*f*_*P*_) move to recovered state (*R*) at rate *γ*_*I*_. Severe infections will be hospitalized for treatment at rate *ω*. Thus, *ω* can be taken as detection rate for infections. A fraction of patients (*f*_*D*_) die at rate *μ* and the remainder of them recover. The dynamics of the model is given by,

1 $$\begin{array}{@{}rcl@{}} \frac{dS}{dt} &=& - \beta(t) S \frac{\theta_{a} A + I + \theta_{p} P}{N}, \\ \frac{dE}{dt} &= & \beta(t) S \frac{\theta_{a} A + I + \theta_{p} P}{N} - \delta E,\\ \frac{dA}{dt} &=& \delta (1- f_{I}) E - \gamma_{A} A, \\ \frac{dI}{dt} &=& \delta f_{I} E - \gamma_{I} I, \\ \frac{dP}{dt} &=& f_{P} \gamma_{I} I-\omega P,\\ \frac{dH}{dt} &=& \omega P -\mu H, \\ \frac{dD}{dt} &=& f_{D}\mu H, \\ \frac{dR}{dt} &=& \gamma_{A} A + (1-f_{P}) \gamma_{I} I + (1-f_{D})\mu H, ~ \end{array} $$

where *N* is the total population size.

The basic reproduction number **R**_0_ is usually estimated in the early stage of the outbreak as a constant value. With the introduction of interventions, the transmission rate *β* is time-varying. Consequently, the reproduction number changes with time, often named as effective reproduciton number and expressed as **R**_*t*_. By linearing the system (Eq. (1)) at the disease free state (*S*,*E*,*A*,*I*,*P*,*H*,*D*,*R*)=(*S*_0_,0,0,0,0,0,0,0) and setting the vector *v*=(*E*,*A*,*I*,*P*)^*T*^, we obtain $\dot {v} = (\mathcal {F}-\mathcal {V})v$, where $\mathcal {F}$ is the new infection rate in each class and $\mathcal {V}$ is the transition rate for each class by transferring in or out of each class, given by

$$\begin{array}{*{20}l} \mathcal{F}&= \left(\begin{array}{cccc} 0 & \theta_{a} \beta(t) & \beta(t) &\theta_{p} \beta(t) \\ 0 & 0 & 0 & 0\\ 0 & 0 & 0 & 0\\ 0 & 0 & 0 & 0 \end{array}\right),\\ \mathcal{V}&= \left(\begin{array}{cccc} \delta & 0 & 0 & 0 \\ -\delta(1-f_{I}) & \gamma_{A} & 0 & 0\\ -\delta f_{I} & 0 & \gamma_{I} & 0\\ 0 & 0 & -f_{P} \gamma_{I} & \omega\\ \end{array}\right), \end{array} $$

from which we can obtain the effective reproduction number of the model (Eq. (1)) as the spectral radius $\rho (\mathcal {F}\mathcal {V}^{-1})$ of the next generation matrix $\mathcal {F}\mathcal {V}^{-1}$. The effective reproduction number **R**_*t*_ is given by

2 $$ ~ \mathbf{R}_{t} = \beta(t) \left(\frac{\theta_{a} (1-f_{I})}{\gamma_{A}}+\frac{f_{I}}{\gamma_{I} }+\frac{\theta_{p} f_{I} f_{P}}{\omega}\right).  $$

Specifically, the basic reproduction number is recovered as $\mathbf {R}_{0}=\beta _{0} \left (\frac {\theta _{a} (1-f_{I})}{\gamma _{A}}+\frac {f_{I}}{\gamma _{I}}+\frac {\theta _{p} f_{I} f_{P}}{\omega }\right)$, where *β*_0_ is the transmission rate in the early stage. The difference between interventions of suppression and mitigation is the aim for affecting the epidemic. Suppression aims at halting the epidemic with extremely strict strategies to satisfy the condition **R**_*t*_<1, while mitigation aims at slowing down the epidemic with relaxed control strategies, resulting in a reduced **R**_*t*_ larger than 1. A strict suppression is more likely to have negative impacts on economics and social lives, while a gentle mitigation may not be able to efficiently control the epidemic. Thus, how to implement an effective intervention strategy to balance the aims is an important and difficult issue to answer.

In order to devise efficient intervention strategies to control epidemics, one has to consider medical resources a city possesses, since infected cases require medical resources for treatment. Also, the timing of interventions being deployed plays a key role in containing epidemic. A delayed, relaxed intervention strategy might be uneffective for controlling the epidemic, since the accumulated existing hospitalized infections may overload the medical resources and further promote the spread of epidemic. Thus, at this stage, a strict suppression strategy is preferentially deployed. This is exactly the strategy some countries like UK adopted. On the other hand, if the existing hospitalized infections are not at risk for medical resources, a gentle, relaxed mitigation strategy would be sufficient enough to control the epidemic.

In the following, we assume that intervention strategies deployed by a city are closely related with its capacity of medical resources *I*_*m*_ and a tolerance level for epidemic *I*_*t*_. At time *t*_0_, if the total hospitalized cases during a period *τ* are higher than the capacity of medical resources *I*_*m*_ with factor *c* (0<*c*≤1), a strict suppression strategy will be implemented; otherwise, if the number of hospitalized cases is higher than some tolerance parameter *I*_*t*_, a mitigation intervention will be deployed. Then, the effective reproduction number **R**_*t*_ is given by

$$ {}\mathbf{R}_{t} \! =\! \left\{\! \begin{aligned} &\alpha_{s} R_{0}, & \ \ \text{if} \sum_{t-(\tau-1)}^{t} H(t) > c I_{m}, t \geq t_{0} \ \ \text{(suppression)} \\ &\alpha_{m} R_{0}, & \ \ \text{if} \sum_{t-(\tau-1)}^{t} H(t) > I_{t}, t \geq t_{0} \ \ \text{(mitigation)}\\ \end{aligned} \right. $$

where *α*_*s*_ and *α*_*m*_ are the mean intensity values associating with suppression and mitigation, respectively, followed by a given distribution [29]. Here, for simplicity, we assume that both *α*_*s*_ and *α*_*m*_ are constants satisfying *α*_*s*_<*α*_*m*_<1. The parameter *I*_*m*_ represents the capacity of medical resources, which may depend on the economical level. For instance, in the US, *I*_*m*_=1.2 beds per thousand people. The parameter *I*_*t*_ represents the tolerance level for epidemic, e.g., $I_{t}=\frac {100}{N}$ means that the tolerance of 100 infections in the population. The relationship between parameters *I*_*m*_ and *I*_*t*_ is tuned by the factor *c*. A value of *c* satisfying *cI*_*m*_>*I*_*t*_ can capture the condition of suppression and mitigation, i.e., *α*_*s*_<*α*_*m*_. Given the parameters for controlling strength, i.e., *α*_*s*_ and *α*_*m*_, and the starting time *t*_0_, the role of interventions is captured by the evolving curve of **R**_*t*_ or *β*(*t*).

It is to note that the deployment of an intervention depends on the number of recently hospitalized cases $\sum _{t-(\tau -1)}^{t} H(t)$, which is related with the accumulated confirmed cases $\sum _{t-(\tau -1)}^{t} I(t)$. In reality, evaluating the exact value of *I*(*t*) is fundamental to understand the impacts of interventions on the epidemic. For the COVID-19, since the real number of infections is generally unknown and only the number of confirmed cases is available, it is necessary to use some approaches to evaluate [29, 33]. Here, in the following, for the real data analysis, we apply a deconvolution method proposed in [29] to estimate the real number of infection cases from the reported infection cases as the initial values of the model. Then, we study the impact of interventions of suppression and mitigation on the epidemic.

## Results

### Modeling results

**Fig. 3:**
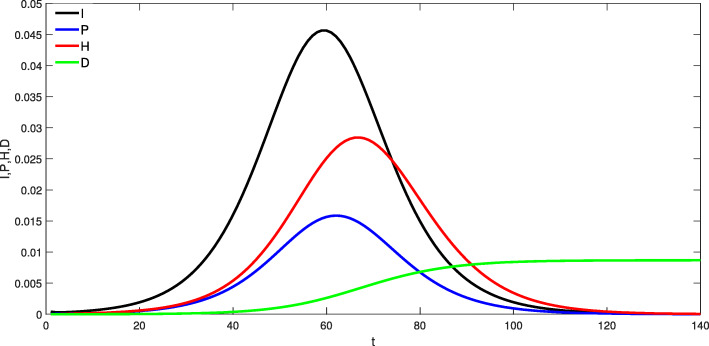
The epidemic dynamics of the model (Eq. (1)) with no interventions. The symptomatic infectious (*I*, black curve), severe infectious (*P*, blue curve), hospitalized infectious (*H*, red curve) and dead (*D*, green curve) are shown in time

We first perform simulations on the proposed model in a host population with no interventions. Then, we compare the effects of interventions such as suppression, mitigation, and the combination of them with the proposed model. The total population is set as *N*=10^6^ and the fractions of exposed individuals and symptomatic infected individuals are *E*(0)=6×10^−4^ and *I*(0)=4×10^−4^, respectively. The basic reproduction number is set as **R**_0_=2.2 for the COVID-19 as Ref. [34]. The incubation period is $\frac {1}{\delta }=5.1$ days [35]. The fraction of symptomatic infections is *f*_*I*_=0.82 as calculated in Ref. [36]. The infectious periods for symptomatic and asymptomatic infection are $\frac {1}{\gamma _{I}}=\frac {1}{\gamma _{A}}=2.3$ days [24]. Since it has been found that there is no difference in the transmission rates between symptomatic and asymptomatic patients [32], we set *θ*_*a*_=1. Usually, the infectiousness of severe infected individuals is higher than that of symptomatic infectious individuals. Here, we assume *θ*_*p*_=1 for simplicity. The fraction of infections requiring hospitalization is set as *f*_*P*_=0.3 and the delay between severe infection and hospitalization is $\frac {1}{\omega }$=2.7 days [24] and the hospital stay is assumed as $\frac {1}{\mu }=8$ days. According to the number of daily deaths and the existing infections in WuHan, the fraction of hospitalized infections to die is calculated as *f*_*D*_=0.065. Without specification, the factor constant *c* is set as *c*=0.8. We did simulations with several values of *c* and found that the choice of *c* does not change the main results of the present study. Most of the parameters are chosen according to the recently published results and some of them are assumed, as summarized in Table 1. Figure 3 illustrates the results for the baseline model, where no interventions are involved. We observe that the infected and hospitalized number reaches the peak at around 50 and 60 days, respectively, approaching 4.5*%* and 3% of the population. The fraction of deaths reaches 0.8*%* of the population, approximately 8000 people.

**Table 1:** Parameters used in the main text

Parameter	Description	Value (range)
*β*(*t*)	Transmission rate with time	$\beta (t) = \frac {\mathbf {R}_{t}}{\left (\frac {\theta _{a}(1-f_{I})}{\gamma _{A}}+\frac {f_{I}}{\gamma _{I}}+\frac {\theta _{p} f_{I} f_{P}}{\omega }\right)}$
**R**_0_	Basic reproduction number	2.2 [34]
$\frac {1}{\delta }$	Incubation period	5.1 days [35]
$\frac {1}{\gamma _{I}}$	The infectious period for symptomatic infection	2.3 days [24]
$\frac {1}{\gamma _{A}}$	The infectious period for asymptomatic infection	2.3 days [assumed]
*f*_*I*_	The proportion of symptomatic infection	0.8 [36]
$\frac {1}{\omega }$	The delay days between severe infection and hospitalization	2.7 days [24]
$\frac {1}{\mu }$	Hospital stay period for severe infection	8 days [24]
*f*_*P*_	The propotion of infection requiring hospitalization	0.3 [24]
*f*_*D*_	The proportion of severe infection to die after hospital stay	0.065[calculated] [31]
*θ*_*a*_	Infectious factor for asymptomatic infections	1.0 [32]
*θ*_*p*_	Infectious factor for severe infections	1.0 [assumed]
*I*_*m*_	Medical resources	0.0023 [calculated] [37]
*I*_*t*_	Tolerance parameter for infection	$\frac {100}{N}$ [assumed]
*α*_*s*_	Suppression coefficient	[0.1,0.4] [assumed]
*α*_*m*_	Mitigation coefficient	[0.5,0.8] [assumed]

#### Effects of suppression intensity *α*_*s*_ and the starting time *t*_0_

**Fig. 4:**
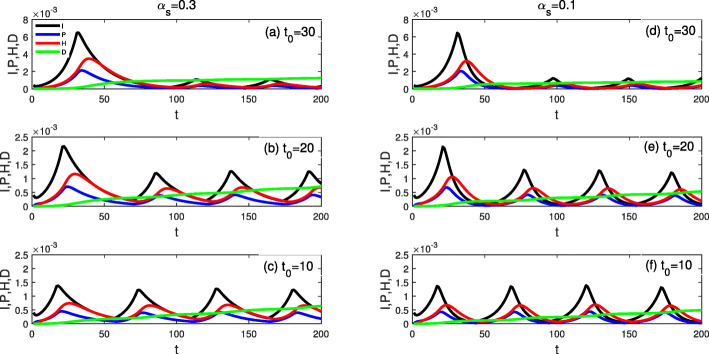
Comparison of the epidemic dynamics under suppression interventions with *α*_*s*_=0.3 (left column) and *α*_*s*_=0.1 (right column) starting at different time. **a** and **d** *t*_0_=30; **b** and **e** *t*_0_=20; **c** and **f** *t*_0_=10. Other parameters are the same as Table 1

In this scenario, we assume that if the fraction of accumulated hospitalization cases during the past *τ* days, e.g., *τ*=7, $\sum _{t-7}^{t}H(t)$, is larger than the medical resources by a factor, *cI*_*m*_, a suppression intervention will be implemented. We take it as a constant control intensity with the average value *α*_*s*_=0.3,0.1 starting at time *t*_0_=10,20,30. These parameters are chosen such that the effective reproduction number **R**_*t*_ is approximatively less than 1. In Fig. 4, we see that with the introduction of a suppression measure with *α*_*s*_=0.3, the peak of infections dramatically reduces to a lower level. For instance, the epidemic peak is reduced by 4.5*%* to 0.7*%* at *t*_0_=30. The earlier the intervention is deployed, the lower the peak of infections will be. If the intervention is deployed 20 days earlier, the infection could be further reduced less than 0.15*%*, see Fig. 4c. Earlier deployment of suppression strategies flatted the curve of dynamics with a significantly reduced peak value and the fraction of hospitalized cases remain at a lower level. The fraction of deaths is also reduced to a lower level due to the extremely efficient intervention of suppression. For instance, at *t*_0_=20, the fraction of deaths is controlled less than 1‰ of the population. In addition, since the introduction of suppression intervention depends on the number of the accumulated hospitalized cases, both the existing infections and the hospitalized infections show a dynamical period of peaks. Earlier deployment of the intervention further brings a more frequent change.

With a more intensive suppression (*α*_*s*_=0.1), the sharp reductions in infections and hospitalizations narrow the curve of the dynamics. In addition, a timely intensive suppression can reduce the deaths to 0.5‰ of the population at *t*_0_=10. Even with a delayed intervention at *t*_0_=30, the deaths can be reduced to approximately 1‰ of the population and the curve of hospitalizations decreases faster. Therefore, to save lives from epidemic, an efficient, strict suppression should be deployed as early as possible. The decreasing slopes of the epidemic curves also provide insightful information for evaluating the effect of different control measures.

#### Effect of mitigation intensity *α*_*m*_ and the starting time *t*_0_

**Fig. 5:**
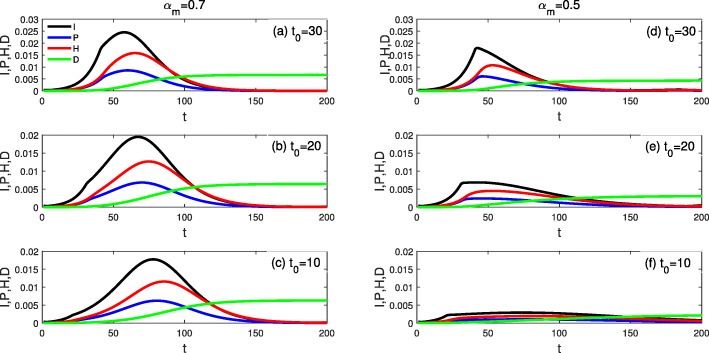
Comparison of the epidemic dynamics under the intervention of mitigation with *α*_*m*_=0.7 (left column) and *α*_*m*_=0.5 (right column) starting at different time. **a** and **d** *t*_0_=30; **b** and **e** *t*_0_=20; **c** and **f** *t*_0_=10. Other parameters are the same as Table 1

Since a suppression intervention is likely to bring negative effects on economics and social activities, a gentle mitigation intervention is feasible to gradually reduce the basic reproduction number **R**_0_. If the ratio of the accumulated existing hospitalized cases during the past week is larger than some tolerance parameter *I*_*t*_, the mitigation intervention is implemented. To compare different mitigation measures, we consider *α*_*m*_=0.7,0.5, evaluating **R**_*t*_=1.54,1.1∈[1,**R**_0_], respectively. In Fig. 5, we see that with a very gentle mitigation *α*_*m*_=0.7, the peak of infections reduces to approximately half of that with no interventions, and the ratio of deaths reduces to 6‰ of the population. Due to the limited role of mitigation intervention in controlling the epidemic, the timing of the intervention does not take obvious effects on the peaks of both infections and deaths. In addition, comparing with the sharp reduction in the curve of the epidemic under suppression intervention, the curve of the epidemic under mitigation further flattens and lasts for longer time. The dynamical period of the peaks of infections is not observed due to the gentle mitigation in slowing down the epidemic.

A more intensive mitigation intervention with *α*_*m*_=0.5 further flatterns the curve of epidemic dynamics with a reduced peak value. The ratio of deaths is also dramatically reduced to less than half of that with no interventions, even starting at late time *t*_0_=30. With a more intensive mitigation, the starting time of the mitigation measure plays a key role in changing the curve of the epidemic dynamics. Earlier interventions can efficiently suppress the epidemic spread and reduce the deaths.

#### A combined intervention of suppression and mitigation

From the above analysis, we find that with an appropriate suppression intervention, the epidemic can be efficiently controlled in short time, while with a gentle mitigation intervention, the epidemic can be slowed down to some degree. To further understand the effects of the two types of interventions on the epidemic, we investigate a strategy of combined intervention of suppression and mitigation. Instead of a manual adjustment of the cycle of the intervention period, we propose a dynamical intervention measure composed of suppression and mitigation depending on the infection level. If the accumulated number of the existing hospitalization cases during the last week is larger than the capacity of medical resources by a factor, then a strict suppression is deployed to suppress the epidemic spread in order to avoid overloading the medical resources. If it is larger than some accepted value, determined by policy-makers, then a gentle, relaxed mitigation intervention is implemented. By doing so, the system can dynamically adjust the interventions depending on the existing hospitalization cases. Based on this assumption, we propose a strategy of combined intervention by setting parameters as *α*_*s*_=0.3 (**R**_*t*_=0.66<1) and *α*_*m*_=0.7 (**R**_*t*_=1.54>1) starting at different time *t*_0_.

**Fig. 6:**
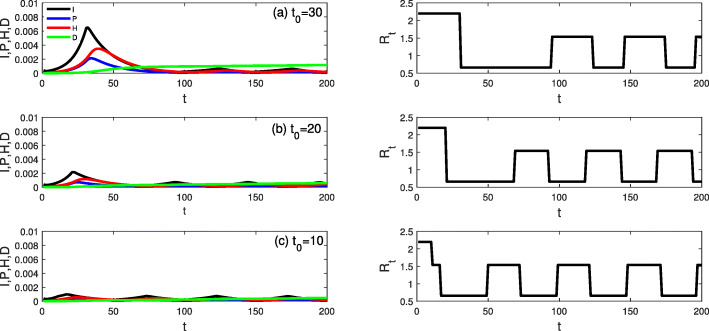
The epidemic dynamics with a combined intervention of suppression and mitigation for different intervention time *t*_0_. **a** *t*_0_=30; **b** *t*_0_=20; **c** *t*_0_=10. The right column corresponds to the effective reproduction number **R**_*t*_ calculated by the intervention strategies. Parameters are set as *α*_*s*_=0.3 and *α*_*m*_=0.7. Other parameters are the same as Table 1

From Fig. 6, we see that when the intervention is deployed at delayed time *t*_0_=30, due to the increased accumulated number of hospitalized infections, a suppression measure lasting for more than one and a half months is necessary in order to keep the epidemic under control, after which a relaxed mitigation measure can follow. Since the deployment of a relaxed mitigation measure may cause the increase of hospitalized infections again, then a strict suppression can be further imposed. Such a periodic iteration of suppression and mitigation proceeds with the hospitalized infections being controlled under a lower level. The period wave for the curve of epidemic dynamics is determined by the intervention time *t*_0_, the medical resources *I*_*m*_ and the tolerance parameter *I*_*t*_. A timely intervention switching between suppression and mitigation will lead to a significant reduction in both the hospitalized infections and deaths.

Our analysis is different from previous study [13], where a period of lockdown or quarantine is fixed manually. By dynamically alternating the intervention with the epidemic dynamics, it is possible to keep the infection under control while allowing a sustainable economy as well as normally social activities.

### Real world data analysis

In the following, we explore the impact of intervention measures on epidemic by analyzing the data of Wuhan for the COVID-19 epidemic. We are aware that the actual infection number is unknown and can only be inferred from other epidemiological observations (e.g., the daily confirmed cases). Such observations are lagging behind the infection events due to inevitable time delays between an individual being infected and reported (e.g., days for symptom onset). In Ref. [29], a deconvolution method is proposed to estimate the actual infection cases from the daily reported confirmed cases with the renewal process. Detailed descriptions of the method refer to Ref. [29]. In this work, we applied this method to infer the actual infection cases on Jan. 11th, 2020. The daily number of onset patients in Wuhan (i.e. daily confirmed cases) is obtained from the study by Pan et al. [38]. Then, we evaluate the infection cases on Jan. 11th as 284. Combined with the information of the ratio of symptomatic infection over asymptomatic infection $\frac {f_{I}}{1-f_{I}}$, the number of asymptomatic infected cases is evaluated as *A*(0)=62. By assuming that the incubation period is 5.1 days, we can get the number of exposed cases on Jan. 11th from the number of onset patients on Jan. 16th as *E*(0)=359. The numbers of recovered cases and death cases on Jan. 11th, 2020 are obtained from [31]. The total population of Wuhan is *N*=11081643. The basic reproduction number is estimated as **R**_0_=2.2 [34] and accordingly, the transmission rate is calculated as *β*_0_=0.7422. With the available data of beds number in Wuhan, the capacity of medical resource of Wuhan is calculated as *I*_*m*_=0.0023 [37]. Other parameters are the same as in Table 1.

**Fig. 7:**
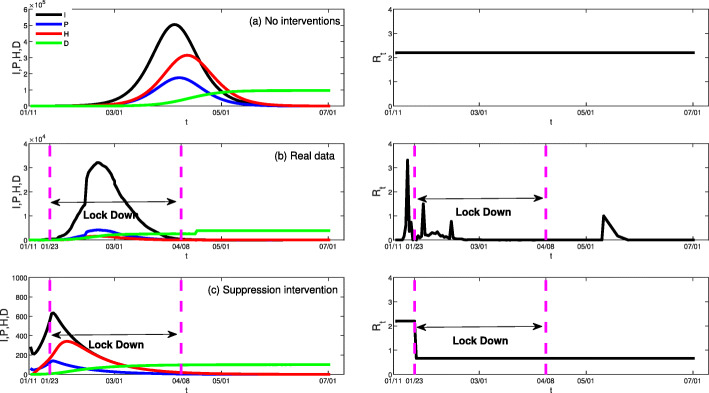
The epidemic dynamics in Wuhan, China from Jan. 11th, 2020 to July 1st, 2020 under different situations. **a** No interventions; **b** real data; **c** suppression intervention with *t*_0_=12 and *α*_*s*_=0.3. The medical resources factor is *c*=1. The lockdown period is indicated by the blue arrow within the dashed lines

Figure 7a shows the epidemic dynamics with no intervention. We see that with no interventions being introduced, it will cause more than ninety thousand deaths in Wuhan. While in reality, as a part of the national emergency response, public transport were suspended, public gathering was banned in Wuhan [10, 39]. The measures taken in Wuhan dramatically reduced the death number less than four thousand by April 17th, 2020 and the peak of infections around thirty-two thousand on Feb. 19th, 2020, as shown in Fig. 7b. It is to note that measures such as lockdown of Wuhan and the construction of mobile cabin hospitals play a key role in suppressing the epidemic, which reduce the reproduction number **R**_*t*_<1 in very short time. To compare the model prediction with the real case, we also perform simulations on the proposed model with the real data. By setting *t*_0_=12 on the lockdown day of Wuhan and *α*_*s*_=0.3 in Fig. 7c, we see that the extremely strict intervention will immediately reduce the reproduction number **R**_*t*_ less than 1 and the deaths will reduce to less than two hundred and the arrival of the peak value will be one month earlier than expected. In reality, the intervention intensity *α*_*s*_ depends on several factors, such as medical resources and the construction of mobile cabin hospitals. The difficulty of obtaining these resources may hinder the control of the epidemic.

**Fig. 8:**
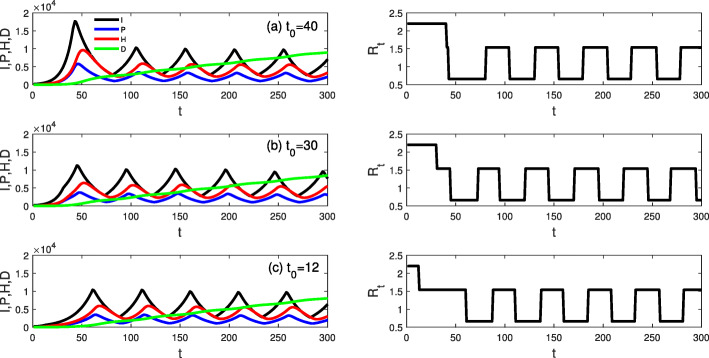
The epidemic dynamics in Wuhan with the combined interventions. **a** *t*_0_=40; **b** *t*_0_=30; **c** *t*_0_=12. The right column shows the effective reproduction number **R**_*t*_ under the combined intervention with *α*_*s*_=0.3 and *α*_*m*_=0.7. The factor for medical resources is *c*=1. Other parameters are the same as Table 1

Next, we investigate what would happen if dynamical intervention were deployed in Wuhan, as shown in Fig. 8. Depending on the intervention time *t*_0_, alternative interventions are dynamically deployed. For instance, with a delayed time *t*_0_=40 (Fig. 8a), the number of infections will increase more than fifteen thousands, consequently, a strict suppression has to be deployed for more than 40 days in order to efficiently control the epidemic, after which a gentle mitigation and a strict suppression alternatively follow. If the intervention start by 10 days earlier (Fig. 8b), the peak value of infections can be reduced to less than fifteen thousands. Then, a shorter suppression followed by a shorter mitigation alternatively controls the epidemic. Finally, if the intervention were deployed at early time with *t*_0_=12 (Fig. 8c), the arrival of the peak value of infections will be delayed and the epidemic is completely controlled periodically.

**Fig. 9:**
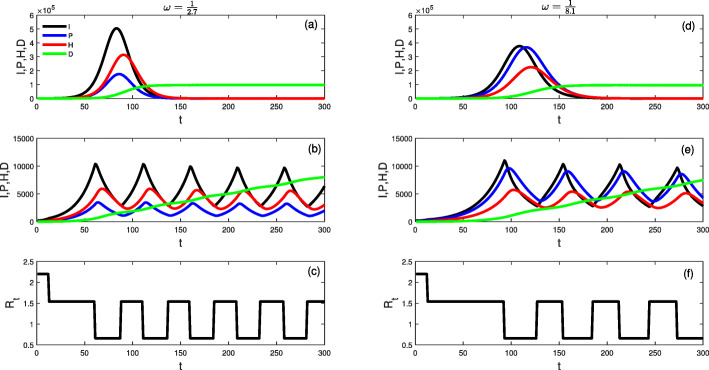
The epidemic dynamics in Wuhan for different choices of *ω*. $\omega =\frac {1}{2.7}$ (left column), and $\frac {1}{8.1}$ (right column) **a** and **d** No interventions; **b** and **e** A combined intervention at *t*_0_=12; **c** and **f** The effective reproduction number *R*_*t*_ under the combined intervention with *α*_*s*_=0.3 and *α*_*m*_=0.7. The factor for medical resources is *c*=1. Other parameters are the same as Table 1

In order to understand the impacts of the detection rate on the epidemic, we carried out simulations with Wuhan data for different choices of *ω* as $\omega =\frac {1}{2.7}, \frac {1}{8.1}$, respectively, in Fig. 9. With no interventions, we see that it will cause less than two hundred thousand severe infections and more than three hundred thousand hospitalized infections (Fig. 9a). With the delay of detection, e.g., $\omega =\frac {1}{8.1}$, the number of severe infections is doubled while the number of the hospitalized infections is reduced. In addition, the arrival of the peak infection will be delayed for one month, as shown in Fig. 9d.

Next, we observe the impacts of the detection rate *ω* on the epidemic dynamics in Wuhan when the combined intervention strategy is involved at *t*_0_=12, as shown in Fig. 9b and e. With a higher detection rate ($\omega =\frac {1}{2.7}$), the number of severe infections reaches less than five thousand and shows a periodical wave under control (Fig. 9b). In such a situation, the intervention measure of suppression frequently alternates with mitigation (Fig. 9c). With the decrease of the detection rate *ω* (e.g., $\omega =\frac {1}{8.1}$), severe infections are hard to be detected and their number is doubled to ten thousand (Fig. 9e). Since the hospitalized infections are decreased, consequently, a longer period of suppression and mitigation is necessary in order to keep the epidemic under control (Fig. 9f).

## Discussion

So far, although several vaccines for the COVID-19 are ready to deploy, non-pharmaceutical interventions are still effective actions to control the COVID-19 pandemic. Therefore, how to implement appropriate interventions for the next stage is fundamentally important for the control of epidemic while sustaining normally social life. It is interesting to point out that extremely strict interventions, such as lockdown and quarantine measures, have substantial effects on changing the epidemic dynamics. With a strict suppression strategy, the number of deaths and that of infections can be reduced to a lower level while long-term measures may have negative impacts on economics and social life.

To solve such a dilemma, we propose a strategy of combined intervention of suppression and mitigation, which dynamically alternates according to the epidemic dynamics. The deployment of suppression interventions is assumed to be relevant with the capacity of medical resources, that is, if the accumulated number of hospitalized infections during a given period is close to the capacity of medical resources with a factor, then a strict suppression will be implemented to avoid overloading medical resources. If the number of hospitalized infections is more than some tolerance level, a relaxed mitigation, such as social distancing and hygiene measures, may be sufficient enough to control the epidemic. Depending on the tolerance parameter, the mitigation strategy can be dynamically switched on or off. Our study shows that early deployment of a suppression intervention will shift the peak value to an earlier date. While timely implement of a mitigation intervention will flatten the epidemic curve with a prolonged period. With a strategy of combined intervention of suppression and mitigation, the epidemic is contained with an acceptable level, where the two measures alternatively interchange with different periods. Such a dynamical deployment of interventions is able to keep the trade-off between economics and epidemic, and take less negative impacts on social life. We believe that such an approach may be adopted as a strategy until the available of effective vaccine.

Our study has certain limitations as well. In reality, the periodic waves of infections in different nations show diverse features, e.g. peaks and periods, depending on how the prevention measures are deployed. Using a simplified Heaviside function alternating between two interventions is not sufficient enough to reflect such a complicated situation in reality. Instead, a non-linear function is expected to offer a solution. Consequently, the present analysis and interpretation of the results are limited to a modeling study for such a phenomenon of periodic waves of infections.

## Conclusions

By analysing the data for countries in battling against the COVID-19 pandemic, we found a periodic-like wave in the number of infections in several countries, which indicates a clear relationship between the infection and the deployed interventions when facing the COVID-19 epidemic. The present study explores the combined impacts of suppression and mitigation measures on the epidemic. The findings are as follows: (a) depending on a city’s capacity of medical resources and its tolerance on infectious population, a combined intervention of suppression and mitigation can efficiently reduce the peak of infections and negative effects on social lives and economics; (b) an immediate, strict suppression measure is highly efficient in reducing the number of infections and brings less losses on people’s lives, as occured in Wuhan, China; (c) a delayed intervention has to be accompanied by a longer suppression followed by a shorter mitigation alternatively, which is expected to balance the loss of people’s lives and that of economics for a long-term control of the COVID-19.

## Data Availability

The number of existing confirmed cases in some countries is available in the DXY-COVID-19-Data Repository (https://github.com/BlankerL/DXY-COVID-19-Data). The data set about daily number of onset patients in Wuhan is available in the https://github.com/nianqiaoju/rt. Declarations

## Abbreviations

COVID-19: Corona Virus Disease 2019
*R*_0_: Basic reproduction number
SEIR model: Susceptible-Exposed-Infected-Recovered model
